# Identification of Mouse Cytomegalovirus Resistance Loci by ENU Mutagenesis

**DOI:** 10.3390/v1030460

**Published:** 2009-10-20

**Authors:** Karine Crozat, Philippe Georgel

**Affiliations:** 1 Department of Genetics, The Scripps Research Institute, La Jolla, CA 92037, USA; 2 Centre d’Immunologie Marseille-Luminy, Université de la Méditerranée, Marseille, F-13288, France; 3 INSERM, U631, Marseille, F-13288, France; 4 CNRS, UMR6102, Marseille, F-13288, France; 5 Faculté de Pharmacie Strasbourg, Illkirch, F-67400, France; E-Mail: pgeorgel@unistra.fr; 6 EA Physiopathologie et Médecine Translationnelle, Laboratoire d’ImmunoGénétique Moléculaire Humaine, Faculté de Médecine, Strasbourg, F-67085, France

**Keywords:** N-ethyl-N-nitrosourea, mutagenesis, resistome, mouse cytomegalovirus, susceptibility, nnate immunity, homeostasis

## Abstract

Host resistance to infection depends on the efficiency with which innate immune responses keep the infectious agent in check. Innate immunity encompasses components with sensing, signaling and effector properties. These elements with non-redundant functions are encoded by a set of host genes, the resistome. Here, we review our findings concerning the resistome. We have screened randomly mutagenized mice for susceptibility to a natural opportunistic pathogen, the mouse cytomegalovirus. We found that some genes with initially no obvious functions in innate immunity may be critical for host survival to infections, falling into a newly defined category of genes of the resistome.

## 1. Introduction

Pathogens represent a constant threat to individuals of almost any multicellular species, and the survival of each such species depends on its ability to resist infections. The immunity of the host may be cell-autonomous, and may also depend upon specialized cells and proteins that collectively comprise the “immune system”. Innate immunity and adaptive immunity are the two main components of host defense in vertebrates. The adaptive immune system depends upon the innate immune system in many ways: its priming requires cytokine signals and antigen acquisition from antigen-presenting cells (APCs), phagocytes of the innate immune system. Therefore, the adaptive immunity cannot exist in the absence of the innate immunity.

The innate immune system reaches full effectiveness rapidly after a pathogen breaches the physical barrier. The rapidity of its reaction may be attributed to the simple transcriptional circuits linking microbial perception to a programmed response. Conserved constituents of microbes are detected by the innate immune recognition receptors, such as Toll-like receptors (TLRs). The response to these microbes, regardless of the stimulus, tends to be stereotyped: the induction of a large number of cytokines such as Tumor Necrosis Factor (TNF), IL-12 or type I interferon (IFN) upon TLR activation for instance, helps to orchestrate the inflammatory response to many different types of bacteria, fungi, and viruses. Cytokines shape the inflammatory milieu, triggering the recruitment and activation of effector cells, leading to the elimination of infectious agents. Every steps of this innate immune response are directed by a set of host genes. The alteration of any of these genes might sometimes be sufficient to favor the spreading of a given pathogen, permitting the host to succumb to an uncontrolled infection. Therefore, susceptibility to infections is largely dependent on the host genome. The set of protein-encoding genes with non-redundant function in resistance to one or more pathogens is the *resistome*.

The number of genes encompassing the resistome is expected to be far fewer than the number of pathogens with which the host must cope. We sought of estimating the size of the resistome of a given microbe, the mouse cytomegalovirus (MCMV), which is a natural infectious agent for mice. The use of random mutagenesis remains the most appropriate way of gradually modifying the host genome, in order to produce phenotype (*i.e.* susceptibility to an infection), and ultimately to determine which genes are required for host protection.

## 2. A Forward Genetic Approach to Unravel the Host Resistome

N-ethyl-N-nitrosourea (ENU) is the most widely and effective germline mutagen used to alter the mouse genome. This alkylating agent is known to induce 0.5 to 1.0 point mutation per million of base pairs [1,2] or 7.5 × 10^–7^ mutations per base pair per gamete of treated male [3], creating one new loss-of-function mutation per gene per 700 gametes [4]. The appearance of a phenotype that can be detected among the ENU-germline mutants after thorough screening, could be ascribed to the alteration of a gene identified by positional cloning.

The size of the host resistome for a defined pathogen may be measured by infecting mutagenized mice and by determining the frequency of phenovariance. MCMV, a viral pathogen with a relatively large genome, was suitable as an infection model and as a screening tool since MCMV tests many aspects of the host innate immune system: elements of microbial sensing, elements of signaling between cells, and elements with effector functions. Therefore, we have defined the *MCMV resistome* as the set of genes with non-redundant function(s) in resistance to MCMV infection in a relatively resistant strain of mouse: C57BL/6J.

This virus infects only mice. It is known that single gene mutations within this host can impair resistance to MCMV and already a handful of genes have been identified as essential for a C57BL/6J mouse to resist MCMV.

## 3. The innate immune response to MCMV infection

The early immune response, which develops during the first days post-inoculation is essential for the rapid clearance of productive viral particles. APCs especially dendritic cells (DCs), and natural killer (NK) cells are the cornerstones of host resistance during this acute phase. MCMV strong tropism for mononuclear phagocytes, such as macrophages or DCs, is beneficial for both the host and the virus. The infection of macrophages and DCs allows a rapid dissemination of the virus in the host. However, macrophages and DCs, when infected, can efficiently detect MCMV initiating a rapid cytokine response [5–7].

### 3.1. Molecular mechanisms of MCMV sensing

As for other pathogens, sensing of the double stranded DNA (dsDNA) virus MCMV requires the recognition of pathogen-derived molecules by members of the TLR family, which are highly expressed in DCs.

TLR3 and its adapter TRIF are presumably involved in sensing dsRNA derived from MCMV genome transcription [8–11], but their contribution is relatively minor compared to that made by TLR9 and its adapter MyD88. TLR9 recognition of the MCMV genome CpG motif was shown to be critical for type I IFN production soon after infection, and for early NK cell activation [9,11,12]. TLR9 signals through MyD88, an adaptor molecule shared by all other TLRs except TLR3, causes NFκB and AP1 activation and subsequent induction of pro-inflammatory cytokines. Interestingly, a deficiency in MyD88 is far more deleterious than a lack of TLR9 in the MCMV-challenged host [11,12] demonstrating the existence of a MyD88-dependent, TLR9-independent sensing pathway. MCMV susceptibility of mice with a combined deficiency in both TLR9 and TLR7, a sensor for single stranded RNA (ssRNA), is reminiscent of the phenotype observed in mice lacking only MyD88 [13]. However, TLR7-deficient mice are not as susceptible as TLR9-deficient mice to MCMV [13] showing that TLR7 contribution in sensing MCMV nucleic acids may complement TLR9 function.

TLR3, TLR7 and TLR9 sensing was found to depend upon a protein called Unc93B1. A deficiency in Unc93B1 causes severe susceptibility to MCMV infection due to a defect in MCMV sensing as demonstrated by a diminished cytokine response in Unc93B1-deficient mice [14]. As in mice, mutations of UNC93B in humans abolish antiviral responses to herpesvirus infection promoting the development of herpes-induced encephalitis [15]. Unc93B1 physically interacts with the endosomal TLR3, TLR7, TLR9 and TLR13 [16] allowing the sorting of these receptors to endolysosomes rapidly after ligand stimulation [17]. This process seems to be TLR-selective since Unc93B1 sequesters TLR7 in the reticulum endoplasmic while preferentially relocalizing TLR9 in endosomes [18] rendering TLR9 more prone to be functional upon stimulation. TLR13, which ligand has not been yet identified may also be involved in nucleic acid sensing and perhaps in MCMV recognition.

Moreover, since TLR3, TLR7 and TLR9 are specifically localized in endosomal compartments in APCs [19,20], and since agents that prevent endosome acidification block TLR3, TLR7 and TLR9 signalling [14], viral nucleic acid sensing by these TLRs requires that the viral DNA must gain access to acidified endosomes. The exact mechanism by which this occurs remains an open question.

### 3.2. Mounting effector functions

Subsets of DCs have been defined according to their cell-surface molecules as well as their properties. The conventional DCs (CD11c^high^ DCs, or cDCs) and plasmacytoid DCs (pDC) subsets are able to detect and efficiently respond to MCMV infection.

#### 3.2.1. The role of pDCs and cDCs in MCMV sensing

pDCs are known for their ability to rapidly secrete large amounts of type I IFN in response to viral infections. In an MCMV-infected C57BL/6J mouse, the induction of type I IFN occurring 36 hours post-infection depends on pDCs and requires TLR9 sensing and MyD88 signaling [11,12], but not TLR7 recognition [13]. However, the role of pDCs in response to MCMV remains unclear since a lack of pDCs does not impair the control of the virus [12,21]. This might be understood on the basis of time-dependent and cell type-dependent production of type I IFN by pDCs. Indeed, 44 hours post-infection, type I IFN production has been shown to occur independently of pDCs [11].

The cDC population is itself infected by MCMV, which interferes with DC functions while actively replicating [5]. Upon infection, cDCs account for the preponderance of IL-12, IL-18 and IL-15 production and also for the late secretion of type I IFN, all of which are essential to achieve full activation of NK cells [22,23]. Among cDCs, the CD8α+ DCs have been identified as essential to maintain the proliferation of activated NK cells during MCMV infection, and in NK cell-depleted mice or in mice deficient in NK cell activation, the disappearance of this DC population is observed [24–26]. It is clear that a strong, mutual dependency exists between CD8α+ DCs and NK cells, but the molecular mechanism of reciprocal activation, which may involve direct cell contacts or cytokine secretion has yet to be defined.

#### 3.2.2. Indirect priming of NK cells by DC-secreted cytokines

Studies of genetically modified mice have revealed which cytokines are essential to mount an effective innate immune response against MCMV. Among all the proinflammatory cytokines secreted by DCs, IL-12, IL-18 and type I IFN are the most important. Each of these cytokines activates specific features of NK cells. IL-12, along with IL-18, is responsible for the induction of IFN-γ in NK cells [27,28]. IL-18 is an ineffective inducer of IFN-γ production by itself, but acts in synergy with IL-12 to optimize IFN-γ secretion by NK cells [25,29]. IL-18 is also required for priming “naïve” NK cells to produce IFN-γ upon IL-12 stimulation [30]. Therefore, a rupture in the signaling axis IL-12/18 **→** IFN-γ as occurring in mice lacking STAT4 [31], a major element in the IL-12 receptor signaling pathway, leads to a failure to clear the virus.

On the other hand, type I IFN is secreted by a wide range of cells at low levels under baseline conditions, and can be rapidly upregulated after viral infection, especially by pDCs as discussed above. Type I IFN binds a unique receptor (IFNAR) and triggers the activation of Jak1 and Tyk2, and consequently a transcription factor composed of STAT1 and STAT2 subunits. IFNAR-deficient mice are highly susceptible to MCMV infection [32] as are mice deficient for molecules involved in the IFNAR pathway [33–36]. Functionally, type I IFN shapes the immune response by directing DC maturation [6], and by inducing IL-15/IL-15Ra complexes on cDCs [23], which not only promotes NK cell blastogenesis [31], but also primes “naïve” NK cell endowing them with cytotoxic activity [22,23,37]. However, as IL-15 and its receptor are essential in NK cell survival, proliferation and homeostasis [31,38,39], mice lacking one of these components are expected to be susceptible to MCMV, and perhaps to succumb rapidly.

### 3.3. NK cells as effectors of the early immune response to MCMV

The effector part of the innate immune response during MCMV infection in both human and mice is ascribed to NK cells only [40–42]. A failure of NK cells to control viremia within a few days following infection is detrimental to the host.

#### 3.3.1. The antiviral activities of IFN-γ

IFN-γ deficiency in mice leads to early lethality after MCMV infection[43,44]. The effect of IFN-γ on target cells reinforces their antiviral states by 1) activating APCs functions[45], 2) enhancing the MHC class I and II-dependent antigen presentation[46] and 3) inhibiting the replication or lytic activity of MCMV[46–48]. The IFN-γ receptor (IFNGR) signals through STAT1, a molecule shared with the IFNAR signaling pathway. Therefore, we might expect that a deficiency in STAT1 is far more deleterious than deficiencies in IFNGR or IFNAR alone.

#### 3.3.2. Elimination of infected cells

Target recognition by NK cell receptors stimulates the remodeling of adhesion molecules at the cell surface in order to form an immunological synapse, whereby cell membranes from both NK cell and target come into close proximity. The formation of this synapse allows the release of highly cytolytic molecules concentrated in NK cell granules. Several molecular components are required for the exocytosis of cytolytic granules. For example, granule tethering to the plasma membrane requires the GTPase activity of Rab27a [49]. However, this exocytic mechanism is shared at least in part by other cell types, such as melanocytes, neutrophils, and platelets. Melanocytes utilize this machinery for the export of granules to the skin or the hair shaft. Therefore, some (though not all) of the genes shown to be involved in exocytosis of lytic granules have also functions in pigmentation, as discussed below.

The release of perforin, a molecule with membraneolytic activity, and granzymes A and B, which initiate the apoptosis of target cells, together account for the final step of the killing of infected cells. No particular granzyme has been found to be essential for resistance to MCMV [50,51], probably due to functional redundancies among members of this family. However, the release of perforin by NK cells is as important as their IFN-γ secretion in the control of the acute phase of MCMV infection [52].

NK cell activation in response to MCMV-infected cells is determined through integration of inhibitory *vs.* activating signals that arise from NK cell receptors and cytokines. Activated NK cells exhibit a specific pattern of migration and proliferation, subsequently leading to the lysis of MCMV-infected cells. Two distinct phases of MCMV-induced NK cell proliferation occur in a C57BL/6J mouse: an early nonspecific proliferation two days post-infection (dpi) is followed at 4 dpi by a preferential expansion of Ly49H+ NK cells, which recognize specifically MCMV-infected cells [53,54].

### 3.4. Sensing of MCMV-infected cells by NK cells

Studies based on quantitative trait loci (QTL) using inbred strains have shown that resistance of mice to MCMV infection is controlled by host genetic make-up with contributions from both major histocompatibility complex (MHC) and non-MHC genes.

#### 3.4.1. The Cmv1 locus

A single locus, designated *Cmv1*, was shown to control MCMV infection independently of the MHC haplotype. Twenty years ago, it was observed that strains of the C57BL background were carriers of a dominant resistance allele (*Cmv1^r^*) whereas susceptible strains (*e.g.*, BALB/c) carried a recessive susceptibility allele (*Cmv1^s^*). The *Cmv1* locus was later linked to the natural killer cell gene complex (NKC) on the distal end of Chromosome 6 [55], and was found to encode Ly49H, an activating NK cell receptor not expressed in the BALB/c strain [56–58]. During MCMV infection, Ly49H recognizes specifically the virally encoded protein m157, which induces activation of Ly49H+ NK cells [59,60]. Ly49H associates principally with the immunoreceptor tyrosine-based activation motif (ITAM) containing adaptor called DAP12 (also known as KARAP). Therefore, mice lacking DAP12 fail to control MCMV infection due to a defect in NK cell activation [54,61,62], and recently DAP10 signaling of Ly49H was found to optimize DAP12 function in NK cells [62]. Its MCMV ligand, m157, encodes a glycoinositol phospholipid (GPI)-linked protein mimicking the structure of MHC class I-like molecules, which normally represent suppressive ligands for proteins of the Ly49 receptor family, most members of which have NK inhibitory functions [60]. The expression of Ly49H by C57BL/6J NK cells is now known to be sufficient to induce resistance exclusively to primary infection with MCMV [63,64].

Although m157 is crucial for Ly49H+ NK cell activation in C57BL/6J mice, Ly49H is rarely expressed in wild mouse populations [65]. It was estimated that 90% of wild mice have been infected by multiple strains of MCMV [66], and most wild isolates of MCMV (∼86%) display mutations in *m157* [67]. Moreover, *m157* has several isoforms that are more or less expressed depending on the cell types infected [68]. This suggests that other recognition mechanisms may determine the outcome of the infection.

#### 3.4.2. The Cmv2, Cmv3 and Cmv4 loci

As the outcome of MCMV infection differs greatly in inbred strains, new *Cmv1*-independent loci have emerged from QTL studies. For example, the two strains New Zealand White (NZW) and New Zealand Black (NZB) possess comparable haplotypes with the C57BL strain on the NKC locus [69]. However, NZB mice are as susceptible as BALB/c upon MCMV challenge whereas NZW mice can control the infection. This mode of resistance was found to be multigenic and associated with loci on Chromosomes 17 and X [70]. These *Cmv2* loci lying outside of the MHC region on Chromosome 17 remain to be identified.

The influence of the MHC class I *H-2* genes on the control of MCMV *in vivo* was initially analyzed using inbred strains and congenic strains. The H-2^k^ haplotype carried by CBA, C3H strains or congenic BALB.K strains was linked to a protective effect against high doses of MCMV [71]. The *Cmv3* locus arose from the H-2^k^ strain MA/My, which displays a *Cmv1*-independent resistance to MCMV [72,73]. QTL analysis has shown that Ly49P, a KARAP/DAP12-associated receptor, can effectively bind H-2D^k^ on MCMV-infected cells in the MA/My strain [73]. The recognition of H-2D^k^ by the NK cell activating receptor Ly49P requires the binding of the MCMV-encoded peptide m04 to the MHC class I niche, which stabilizes the expression of MHC class I complexes at the surface of infected cells [74]. Although the expression of H-2D^k^ molecules at the cell surface is downregulated by MCMV [75], the Ly49P recognition of H-2D^k^/m04 complexes seems to account for the NK cell-mediated viral control observed in the MA/My strain [74].

An additional report has highlighted the existence of a new resistance mechanism independent of the m157-Ly49H interaction. The wild-derived inbred strain PWK/Pas controls MCMV infection identically to the C57BL/6J strain with a minor contribution of *H-2* genes and despite the lack of Ly49H expression. The *Cmv4* locus, mapped to the NKC region on Chromosome 6, might encode a new NK cell activating receptor that enables the detection of MCMV-infected cells as does Ly49H in the C57BL strain [76].

Sensing components (*i.e.* TLRs, activating NK cell receptors) and signaling components (*i.e.* cytokines, transmitters), of the innate immune system aim at mounting effector functions provided mainly by NK cells in MCMV infection.

## 5. Screening for resistance loci

The strong phenotypic difference between C57BL/6J and BALB/c strains, with respect to MCMV resistance, is well understood to be monogenic. The optimal dose of virus used to screen ENU-germline mutants generated on a pure C57BL/6J background was chosen because it would readily discriminate between the C57BL/6J strain and the BALB/c strain, and as such, discriminate between different alleles at the *Cmv1* locus. It was reasoned that all mutations exerting a phenotypic effect as strong as or stronger than that caused by deletion of the *Ly49h* gene would be identified in the screen.

In this screen, a total of 3,500 ENU-germline mutants—corresponding to 583 pedigrees—have been infected with MCMV, and 11 potential susceptible mice were recovered. After further generation testing, three pedigrees were considered to be false positives and discarded, while 8 mutations were transmissible. Among these 8 mutations, 6 have been identified, one remained to be mapped, and one has been lost due to a poor breeding. This method of screening was validated by the characterization of *Domino*, the first susceptibility phenotype [36].

### 5.1. Is Domino susceptibility linked to an alteration of STAT1 configuration?

The *Domino* mutation was not ascribed to a Chromosome location by positional cloning, but rather deducted by hypothesis. *Domino* mutants were more susceptible than BALB/c mice to MCMV infection, showing lethality at day 4 post-infection, before BALB/c controls. It was also observed that *Domino* peritoneal macrophages could not control the replication of the RNA virus Vesicular Stomatitis virus (VSV) *in vitro*. Since resistance to VSV infection is mostly mediated by the action of type I IFN [77], and since *Domino* macrophages were insensitive to IFN-γ treatment while infected with VSV, the *Domino* phenotype was linked to a defect in type I IFN receptor (IFNAR) itself or its pathway. Sequencing of all molecular components involved in the type I IFN signaling has revealed a missense mutation in STAT1 affecting its function in *Domino* mutants [36].

Heterodimers of STAT1/STAT2 and homodimers of STAT1 are essential for the signal transductions of type I and type II (IFN-γ) IFNs respectively. During the acute infection by MCMV, type I and type II IFNs confer non overlapping protective functions. As explained above, type I IFN is produced by pDCs mainly in response to TLR9/MyD88 signaling, and activates NK cell cytotoxic functions. Type II IFN is produced chiefly by activated NK cells, and reinforces the antiviral state of macrophages and DCs. At the structural level, the *Domino* mutation affects a protuberant amino acid in the DNA binding domain of STAT1. Non-phosphorylated STAT1 molecules remain as dimers in the cytosol [78], adopting either a “parallel” or an “antiparallel” structure. In both structural models, the DNA binding domain interacts with other domains stabilizing STAT1 dimers [79,80]. Because this domain is involved in both possible configurations of STAT1 dimers (phosphorylated and non-phosphorylated), cytosolic STAT1*^Dom^* dimers might not be stable as shown by the relative decrease in the total amount of STAT1 protein in *Domino* macrophages [36]. STAT1*^Dom^* might also fail to dimerize with STAT2 upon type I IFN stimulation since different members of the STAT family display closely similar configurations [81].

*Domino* has a practical utility since it is the first mutation in STAT1 identified on a pure C57BL/6J background. This mutation is interesting from a structural point of view since it is still not clear why a defect in the DNA binding domain of STAT1 has such strong effect on the phosphorylation state of the protein.

### 5.2. Jinx: a step forward in the understanding of a human disease

The *Jinx* phenotype was identified in an ENU mutagenized mouse that became severely ill following inoculation with MCMV. When fixed in a homozygous stock, the mice developed higher-than-normal cytokine levels following infection, and higher-than-normal viral burden, consistent with an effector defect rather than a sensing or a signaling defect. As opposed to *Domino* mutants, *Jinx* susceptibility to MCMV was exclusively associated with an absence of NK cell cytolytic activity [82]. For example, the *Jinx* mutation did not cause susceptibility to VSV in cultured cells *in vitro. Jinx* NK cells fail to degranulate, a deficit also observed in activated CD8 T cells [82]. The *Jinx* phenotype was attributed to disruption of *Unc13d* gene by a splicing error. *Unc13d* is the mouse homologue of the human MUNC13-4. MUNC13-4 is known to prime cytolytic granules, rendering them competent to fuse to the plasma membrane of NK and CD8 T cells, and mutations affecting this gene are linked to the development of the subtype 3 of familial hemophagocytic lymphohistiocytosis (FHL), a genetic form of hemophagocytic lymphohistiocytosis (HLH) [83–85]. In human, it was suggested although not certain that infectious agents were the triggering factors of HLH. In our model, neither infections with low dose of MCMV nor inoculation with *Listeria monocytogenes* trigger HLH-like symptoms in *Jinx* mice. In these two cases, *Jinx* mice could control the infectious agent within 14 days following inoculation. However, when infected with the clone Armstrong of the lymphocytic choriomeningitis virus (LCMV), known to induce strong CD8 T cell responses leading to a rapid control of the infection in wild-type mice, *Jinx* mice develop clinical features resembling HLH-like disease. Twelve days post-inoculation, while wild-type mice have cleared LCMV infection, *Jinx* mice are still affected by an overproduction of IFN-γ, an overwhelming CD8 T cell proliferation and activation, an overactivation of macrophages (hemophagocytosis), severe organ infiltrations of immune cells, and fail to control LCMV.

**Figure 1.**
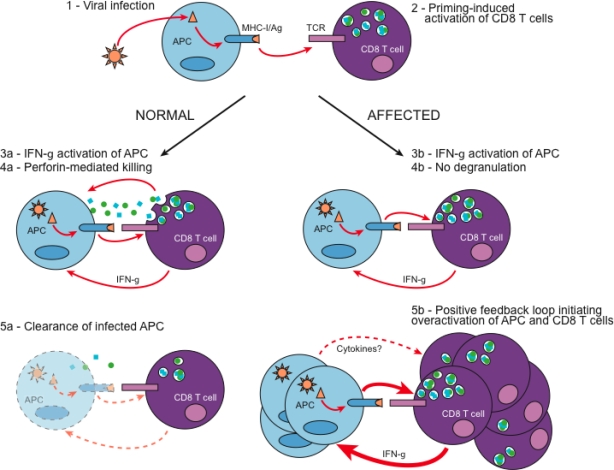
Hypothetical model explaining the development of HLH-like disease in *Jinx* upon LCMV infection. **(1)** The murine RNA virus LCMV Armstrong strain infects preferentially APCs, especially macrophages and few DCs. **(2)** Infected APCs prime the proliferation and activation of LCMV-specific CD8 T cells. In normal individuals, activated LCMV-specific CD8 T cells produce IFN-γ, which in turn promotes APC maturation **(3a)**. The release of granzymes and perforin by activated CD8 T cells is crucial for the rapid elimination of LCMV-infected APCs **(4a)**, and subsequent viral clearance in the host **(5a)**. In individuals with a defect in CD8 T cell cytotoxicity, although IFN-γ is produced in response to infection **(3b)**, infected APCs are not eliminated probably due to either a defect in degranulation as occurs in *Jinx* mutant **(4b)**, or a defect in the cytolytic activity of perforin. The persistence of LCMV-infected APCs, which maturation increases as IFN-γ is secreted, amplifies CD8 T cell activation, effector functions and proliferation. Therefore, in our model, a lack of degranulation of CD8 T cells promotes a positive regulatory loop between APCs and CD8 T cells initiating severe immunopathology reminiscent of HLH disease **(5b)**.

Our study of LCMV-infected *Unc13d^Jinx^* mice contributed in defining the major mechanisms underlying the development of HLH. In a normal host, LCMV-infected APCs present LCMV-derived antigens to CD8 T cells. CD8 T cells respond by IFN-γ production, and degranulation of perforin and granzymes that kill LCMV-infected cells in their close vicinity (Figure 1). The activation state of CD8 T cells correlates with the control of the viremia, until infected cells in the host are cleared. In *Jinx*, LCMV-activated CD8 T cells fail to degranulate allowing the persistence of LCMV-infected APCs, and further activation of CD8 T cells. In the same time, overactivated CD8 T cells produce larger amounts of IFN-γ. IFN-γ may contribute in maintaining the CD8 T cell priming capacity of LCMV-infected APCs, and perhaps may act directly on CD8 T cells increasing their proliferation [86]. Genetic inactivation of IFN-γ signaling in *Jinx* mice prevents the development of HLH-like symptoms upon LCMV infection, but increases their viral load in both liver and spleen (unpublished data). This supports that a positive regulatory loop between APCs and CD8 T cells may be sufficient to rapidly elicit the appearance of clinical features of HLH-like disease as described in human (Figure 1).

#### 5.2.1. Is the function of Unc13d cell-specific?

Some of the genetic alterations resulting in degranulation defects also result in hypopigmentation: *Ashen* (*Rab27a*) and *Beige* (*Lyst*) mice are models for human Griscelli syndrome type II and Chediak-Higashi syndrome respectively, and in each case, melanosome exocytosis is impaired. In human as in mice, these syndromes are associated with the development of HLH-like diseases [87–89]. In other mouse models such as *Gunmetal* (*Rabggta*) and *Pearl* (*Ap3b1*), vesicle trafficking in melanocytes and in hematopoietic cells is also affected, but no occurrence of HLH disease has been reported, probably because such mutations affect only partially the exocytosis machinery. The expression of these genes (*Rab27a*, *Lyst*, *Rabggta* and *Ap3b1*) is not only confined to NK or CD8 T cells; rather protein expression could be detected in neutrophils, basophils, mast cells or platelets. For instance, all of these four coat color mutants display prolonged bleeding times caused by reduced numbers of dense granules in platelets [90–94].

In contrast, the *Jinx* mutation does not cause a defect neither in pigmentation, nor in platelet function as demonstrated by normal bleeding time test (unpublished data). In neutrophils, MUNC13-4, the *Unc13d* gene product, is required for the exocytosis of azurophilic granules containing inflammatory factors and myeloperoxidase, and specific granules containing immunomodulators upon stimulation [95]. At steady state, MUNC13-4 is mainly found in the cytoplasm of neutrophils. After activation, MUNC13-4 is rapidly recruited at the plasma membrane in a calcium-dependent manner, and primes granules *via* the binding to phospholipids of vesicles [96]. Although other cell types have to be analyzed, these observations suggest that Unc13d is chiefly required in immune cells.

#### 5.2.2. Is FHL-2 distinguishable from FHL-3?

*PERFORIN-1* is mutated in patients affected by the subtype 2 of FHL (FHL-2) [97] and perforin-deficient mice develop HLH-like syndrome after viral infection [98]. Studies have indicated that Fas ligand (Fas-L) localizes specifically in secretory lysosomes in CD8 T cells and NK cells, and that degranulation is essential for Fas-L cell surface expression [99,100]. Since *Jinx* and perforin-deficient NK and CD8 T cells are distinguishable by their capacity to degranulate, one may ask whether the HLH-like disease in *Jinx* is more severe than in perforin-deficient mice, which can express surface Fas-L. Fas-L expressed at the immunological synapse, would, at least potentially, offer another route through which effector function could be achieved.

*Jinx* mice provide good model for 1) studying the effect of a lack of NK cell and/or CD8 T cell functions, 2) exploring the pathogenesis of FHL-3 in MUNC13-4-deficient patients and 3) identifying the consecutive molecular steps of the exocytosis mechanism.

### 5.3. Warmflash and Moneypennie mutants: new susceptibility phenotypes?

*Warmflash* and *Moneypennie* mutants are distinct from *Domino* or *Jinx* mutants as they do not constantly display a high viral burden in their spleens similar to that in BALB/c spleens on day 5 post-infection. However, they both fail to clear MCMV in the liver (unpublished data).

*Warmflash* mutants die within 5 and 6 days after challenge with twice the dose of MCMV used for the screen. The induction of cytokines in response to infection is minimally affected in comparison with MCMV-infected C57BL/6J mice. Studies on *Warmflash* peritoneal macrophages did not detect any defect in sensing nor susceptibility to VSV. Despite an apparent reduced number of NK cells *in vivo*, *Warmflash* NK cells respond normally to cytokine and NK cell receptor stimulation *in vitro*. Preliminary results on the positional cloning of this mutation suggest that *Warmflash* susceptibility may be associated with an immune mechanism not fully explored yet. In fact, it was observed that *Warmflash* mutants display lower number of splenocytes at steady state, and the size of their spleens remain normal after MCMV inoculation suggesting a failure to respond to the infection. On the other hand, *Moneypennie* mutants produce higher levels of type I IFN and IFN-γ than C57BL/6J controls after MCMV infection, and no NK cell defect has been reported *in vivo*.

Which host immune mechanism may permit a faster clearance of virus in the spleen than in the liver? Controversies have arose when two studies have shown that NK cells regulate MCMV infection *via* production of perforin in the spleen and IFN-γ in the liver [28,101] whereas other groups have presented different results pointing to an IFN-γ-independent cytotoxicity-dependent mechanism in the liver [102]. These differences could be explained by the mixed genetic background of mice used for each of these studies. Indeed, it was found that NK cells utilize both perforin- and IFN-γ-dependent mechanisms to regulate the acute phase of MCMV infection both in the spleen and the liver in C57BL/6J mice [52]. However, we couldn’t exclude the existence of a compartmentalization of the immune response in the context of MCMV. For example, it was observed that, whereas IL-12 is essential to induce IFN-γ in both the spleen and the liver, IL-18 is not required for IFN-γ induction in the liver in response to MCMV [29]. In contrast, the chemokine MIP-1α was found essential for an IFN-γ response in the liver but not in the spleen [43]. Screening for MCMV susceptibility mutations may as well give an insight about the disparate outcome of MCMV replication in these organs.

### 5.4. The MayDay phenotype: unraveling the importance of homeostasis in host survival

Among the mutants recovered from the screen for MCMV susceptibility, four displayed common characteristics with respect to the timing and mode of death following infection. These four mutants, called *MayDay*, *Solitaire*, *Goodnight* and *Slumber*, showed enhanced lethality 2 to 3 days after infection [36,103]. In these mutants, death occurred abruptly before high viral titers could be achieved, and in all cases, the peak of cytokine response, measured 36 hours post-inoculation, was minimally affected [103]. Since these mutants die within the range of time during which cytokine production peaks in response to MCMV infection [104], proinflammatory cytokines rather than MCMV cytopathic effects might account for their susceptibility. The *Slumber* mutation was mapped to the distal end of Chromosome 6, encompassing the critical region for *MayDay*, and complementation tests revealed that all four of the mutations were allelic [103]. Their abrupt death following MCMV challenge was also observed after lipopolysaccharide (LPS) administration, *Listeria* infection or unmethylated DNA bearing CpG motifs (CpG) administration. The *MayDay* defect is not intrinsic to the hematopoietic system, and is rather attributed to a lack of vasodilatory responsiveness of coronary vessels in response to cytokines and/or metabolic stress. In fact, a complex rearrangement of the *Kcnj8* locus is responsible for the conditional lethality observed in *MayDay* mutants. *Kcnj8* encodes the potassium channel protein Kir6.1 (inwardly rectifying K^+^ channel 6.1), which associates with the sulfonylurea receptor SUR2 to form an ATP-sensitive potassium (KATP) channel. Kir6.1 expression is restricted to smooth muscle cells in the coronary arteries [105], strongly suggesting that a lack of physiological response causes severe myocardial ischemia and infarction leading to the death of the host. Interestingly, Kir6.1 function in maintaining the host homeostatic state during innate immune responses to infections seems to be conserved among species. In *Drosophila*, the KATP channel consists of two Kir6.1 homologs and one sulfonylurea receptor ortholog, dSUR. Flies with a reduced expression of dSUR in the heart are hypersusceptible to the RNA virus Flock House virus (FHV) compared to control flies. However, a defective expression of dSUR is not deleterious for flies infected with the Drosophila C virus (DCV), enterobacteria or with the fungus *Beauveria bassiana* [103]. Perhaps infection with FHV induces a state of hypoxic stress with which dSUR-deficient *Drosophila* could not cope. In contrary, infection with DCV, bacteria or fungi may not induce any septic shock-like effects.

The role of Kir6.1 in regulating the host homeostasis upon infection is based from the analysis of the 4 allelic mutants *slumber*, *goodnight*, *mayday* and *solitaire* isolated from 4 different unrelated ENU-mutagenized pedigrees. The genetic alteration affecting Kir6.1 may not have arisen from ENU mutagenesis *per se*. but may have likely occurred in the C57BL/6J stock of mice prior to ENU treatment.

Nonetheless, this example highlights the fact that the ability of the host to survive an infection depends not only on innate immune mechanisms, but also upon homeostatic mechanisms that permit survival in the context of an innate immune response. It is possible that inter-individual differences in the efficacy of such homeostatic mechanisms largely determine who will live and who will die as a result of infection.

## 6. The MCMV resistome

As reported several times before [36,106,107], we sought to mathematically calculate the size of the MCMV resistome. The *genomic footprint* of a phenotype refers to the set of nucleotides, spread across the genome that can yield the phenotype in question when altered by mutation [2]. Recently, we have estimated to ∼34,200 bp the total number of nucleotide targets that can lead to MCMV susceptibility when mutated. These nucleotides are parceled among a number of genes of the MCMV resistome that we have estimated to ∼321 genes (Crozat K., Beutler B., unpublished data).

In common experience with ENU, phenotype results from changes in coding sense. The nucleotides that comprise the genomic footprint of a phenotype are therefore parceled among unknown number of genes. The genomic footprint and the MCMV resistome itself are gradually saturated in the course of ENU mutagenesis, ultimately leading to *phenotypic saturation*, wherein all genes that can support a phenotypic change through alteration have been identified. So far, the mammalian genome has not been saturated to a level that would permit the precise description or distribution of the target sizes of the MCMV resistome. Our current method of screening will closely approach to a certain level the exhaustion of these genes.

Some of the genes of the MCMV resistome contribute to the development of the immune response leading to host resistance. The sequential mechanisms of this response have different functions that can be categorized (Table 1). In one category, belong genes dedicated to sensing viral infection (genes with sensing functions). In a second category belong genes with roles in post-sensing mechanisms. Among genes encoding proteins with sensing functions, *Ly49h* and *Tlr9* are the most used by host to detect MCMV. Signal transducers or transmitters (e.g., *Dap12*, *Myd88*, *Stat1*) and signaling molecules (e.g., *Il12*, *Ifna* and *Ifnb*) serve as links between sensing and post-sensing functions. Post-sensing mechanisms are encoded by genes that shape cells to become fully functional (maturation, proliferation, trafficking) and by genes that encode proteins with effector functions (e.g., *Unc13d*, *perforin*).

Even if some of the mechanisms have been already described, many other genes remain to be identified in these two categories. New sensors of MCMV-infected cells are sporadically identified as *Cmv-1*-independent loci encoding for NK cell receptors [70,73,74,76] suggesting that sensing mechanisms, and other mechanisms required in interactions between cells and certainly in effector functions, are not fully understood yet. For instance, important roles for cellular non coding microRNAs (miR) in immune responses to Herpesviruses (HCMV) have been recently reported [108]. Additional mutants affecting the generation and processing of cellular or virally-encoded non coding RNAs playing essential effector functions might as well be uncovered in future ENU-based screens designed to identify phenodeviants.

Using this screen, we have identified the *MayDay* mutation altering a gene that cannot be placed in any of the former categories. This gene, involved in maintaining physiological homeostasis of the host, may be part of a new category of the MCMV resistome called the “stabilizing” or the “homeostatic category”. Genes in this category are required for the host to survive inflammation, and especially inflammation-induced stress. Another essential physiologic adaptation that occurs in the course of MCMV infection is seen in the role of endogenous glucocorticoids, which guard against cytokine-mediated lethality [104,112]. Therefore, we believe the size of this set of genes is still underestimated. Stabilizing genes are expected to be rather abundant in the host genome because they are required for many different metabolic pathways and indispensable physiological processes. They might be required at each level of the immune response, from within a few minutes of pathogen entry to the completion of the inflammatory response. They might also be essential for host survival during many infections, not only to MCMV, and might therefore belong to the “global” host resistome.

The “general” host resistome encompasses components, which functions in host resistance are ancient and conserved from invertebrates to vertebrates. This is the case for “host homeostasis” components as seen above, but also for genes with functions in immune responses. Sensing by Toll receptors and cell signaling by type I IFN are such examples: *Drosophila*, as mammals, use Toll and JAK/STAT signaling upon infection. Some of the TLRs (e.g., TLR9) and their adapters (e.g., MyD88 and Unc93B1) are likely to confer protection to a large panel of microbes in mammals.

Some resistance mechanisms are considered as “collective” because they apply to a wide range of microbes, but not all, within a given species (e.g., LCMV and MCMV, Vesicular Stomatitis virus (VSV), and other viruses). For example, some mutations that cause susceptibility to MCMV may also cause susceptibility to LCMV as observed in *Jinx* mice [51,82,98]. Other mutations that cause susceptibility to MCMV and LCMV may also cause susceptibility to VSV [36]. This underlines a lack of specificity in the innate immune responses to viruses.

**Figure 2.**
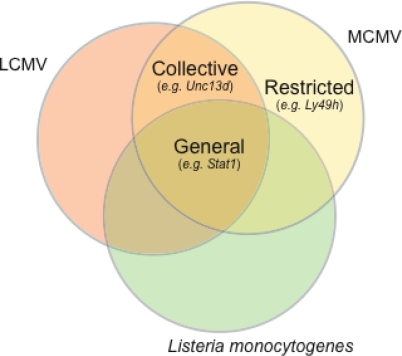
Overlapping resistomes and definition of general, collective and restricted resistomes. Genes involved in the host resistance to a wide range of pathogens belong to the so-called “general resistome”. For example, the *Stat1* gene with unique function in type I IFN and IFN-γ signaling pathways is required for the host to resist viruses (e.g. the RNA virus LCMV, the DNA virus MCMV) and bacteria (e.g. *Listeria monocytogenes*). Other genes with critical functions in the immune responses to viruses, and not bacteria, (e.g*. Unc13d*) belong to the “collective resistome”, whereas genes with critical functions in the immune responses to a unique pathogen (e.g. *Ly49h*, see text) belong to the “restricted resistome”.

Other mechanisms of resistance are highly specific to a pathogen, and may exist in only one species to which the pathogen has adapted. This set of resistance mechanisms belongs to the “restricted” mechanisms of the mouse (Figure 2). For example, *Ly49h* gene is expressed in the MCMV-resistant strain C57BL/6J, and has a highly specific role in the sensing of MCMV. To date, Ly49H has no role in the sensing of pathogens other than MCMV, and a lack of Ly49H has no known immune consequences other than susceptibility to MCMV [63,64].

Certainly, genes identified in the MCMV resistome may fall into all three mechanisms defined here as “general”, “collective” and “restricted”. As these genes provide resistance to MCMV infection, other genes may have “latent” functions in resistance. This state of resistance could be threatened by mutations. For example, C57BL/6J mice generally die within few days after inoculation with 10^6^ pfu of MCMV, because the immune system is insufficient to cope with the overwhelming quantity of replicative viral particles. With such a dose, susceptibility is certainly triggered by host genes permitting the virus to enter the cells and to replicate extensively. The set of host genes with non-redundant functions that allow MCMV replication in a C57BL/6J mouse define the MCMV *susceptome*. A loss of function of one of these genes will be sufficient to completely block virus survival in the host. To date, the only reported examples of increased resistance to MCMV infection concerns cells that have undergone inhibition of members of the protein kinases C (PKC) family [113], and mice deficient for the methylenetetrahydrofolate reductase (MTHFR) [114], which are both required independently for the MCMV replication From the standpoint of clinical research, this screen is well adapted for development of therapies to fight human CMV. So far, only a few cytomegalovirus vaccines have advanced to the stage of efficacy testing [115]. A 6-week course of ganciclovir given intravenously to neonates is the only method to stop progression of CMV infection and its related disabilities [116].

## Figures and Tables

**Table 1.** Genes required for the innate immune response to MCMV infection.

	**Gene name**	**Protein**	**Evidence [References]**
**Sensors**	*Ly49h*	Ly49H	QTL [56,57]
	*Tlr3*	TLR3	KO [9]
	*Tlr9*	TLR9	ENU [9]
	*Il15rb*	IL-15R	Blocking Ab [31]
	*Ifngr*	IFN-γR	KO [109]
	*Ifnar*	IFN-αR	KO [32]
**Extracellular signals**	*Il12b*	IL-12	KO [29,43]
	*Il18*	IL-18	KO [29,43]
	*Infg*	IFN-γ	KO [43]
	*Mip1a*	MIP-1α	KO [110]
**Transmitters**	*Dap12*	KARAP/DAP12	KO [61,62]
	*Myd88*	MyD88	KO [9,11,13]
	*Trif*	Trif	ENU [8]
	*Irf-1*	IRF1	KO [109]
	*Stat4*	STAT4	KO [31]
	*Stat1*	STAT1	KO [33,34]
	*Tyk2*	TYK2	KO [35]
**Effectors**	*Pfr1*	Perforin	KO [52, 101,102]
	*Lyst*	Lyst	QTL [111]
	*Rab27a*	Rab27a	QTL (Georgel P.,
	*Unc13d*	Unc13d	ENU [82]
	*Inos*	iNos	KO [109]
**Homeostasis**	*Kcnj8*	Kir6.1	ENU? [103]

*KO*, knock-out, *Ab*, antibody, *QTL*, quantitative trait loci, *ENU*, N-ethyl-N-nitrosourea.
